# Assessment of a rapid diagnostic test to exclude bacteraemia and effect on clinical decision-making for antimicrobial therapy

**DOI:** 10.1038/s41598-020-60072-9

**Published:** 2020-02-20

**Authors:** Samuel Yui, Georgia Bercades, Monika Muzslay, Emma Blackburn, Shanom Ali, Deborah Smyth, Alison Macklin, Jung Hyun Ryu, Paul Bassett, Niall MacCallum, David Brealey, Peter Wilson

**Affiliations:** 1 Environmental Research Laboratory, University College London Hospitals, 235 Euston Road, London, NW1 2BU UK; 2 Division of Critical Care, University College London Hospitals, 235 Euston Road, London, NW1 2BU UK; 3 Statsconsultancy Ltd, Amersham, Bucks UK; 4 Clinical Microbiology & Virology, University College London Hospitals, 235 Euston Road, London, NW1 2BU UK

**Keywords:** Infectious-disease diagnostics, Laboratory techniques and procedures, Outcomes research

## Abstract

Unnecessary antimicrobial treatment promotes the emergence of resistance. Early confirmation that a blood culture is negative could shorten antibiotic courses. The Cognitor Minus test, performed on blood culture samples after 12 hours incubation has a negative predictive value (NPV) of 99.5%. The aim of this study was to determine if earlier confirmation of negative blood culture result would shorten antibiotic treatment. Paired blood cultures were taken in the Critical Care Unit at a teaching hospital. The Cognitor Minus test was performed on one set >12 hours incubation but results kept blind. Clinicians were asked after 24 and 48 hours whether a result excluding bacteraemia or fungaemia would affect decisions to continue or stop antimicrobial treatment. Over 6 months, 125 patients were enrolled. The median time from start of incubation to Cognitor Minus test was 27.1 hours. When compared to 5 day blood culture results from both the control and test samples, Cognitor Minus gave NPVs of 99% and 100% respectively. Test results would have reduced antibiotic treatment in 14% (17/119) of patients at 24 and 48 hours (24% at either time) compared with routine blood culture. The availability of rapid tests to exclude bacteraemia may be of benefit in antimicrobial stewardship.

## Introduction

The emergence of multi-resistant pathogens is a major health concern^1^. Antimicrobial stewardship is the main defence against these organisms and shorter courses of antibiotic are required. The earlier that antibiotic treatment can be safely stopped the less the pressure on emergence of resistance and the longer the potential life of the antibiotics that are active against hospital pathogens. In critical care, the microbiologist and the clinician discuss antimicrobial treatment daily. Antibiotics will be stopped when clinical signs and inflammatory markers indicate they are not needed but, where there is uncertainty, the antimicrobial course is continued at least until the fifth day^2^. The presence of fever, raised white cell count (WBC) and/or C-reactive protein (CRP) and inotrope requirement as well as local signs determine how long antibiotic treatment is continued but none of these are specific to the presence of infection^3^.

Stopping of antibiotic earlier could be promoted by a test that could exclude the presence of organisms in the first blood culture. Positive blood culture results are usually available at 24 hours but negative cultures are not confirmed until 5 days. The presence of antibiotic or a fastidious organism can delay detection of growth. In some patients the resolution of signs alone may be insufficient to suggest stopping antibiotics.

This study examines the performance and potential clinical effect of a diagnostic test that excludes the presence of bacteria and fungi in blood specimens. This information would be available the day after collection and this study assesses any influence on the decision to discontinue antibiotic treatment. The Cognitor Minus test (Momentum Bioscience Ltd., Long Hanborough, UK) is performed on negative blood samples >12 hours incubation. Using Enzymatic Template Generation and Amplification (EGTA) technology, the test detects microbial DNA polymerase activity common to a wide range of bacteria and fungi^4^. The test has a negative predictive value of 99.5%^5^. When a sample cannot be confirmed as negative, the result is reported as ‘Not Determined’; this would be expected to include positive cultures, bacterial growth insufficient to trigger positive result in the culture system, viable but non-culturable organisms, antibiotic-damaged, autolysing or slow growing organisms as well as inherent false positive results.

## Methods

The aim was to determine if early provision of a negative blood culture result would reduce the length of antimicrobial treatment and whether removal of a sample to test would affect rates of contamination. Any patient on the intensive care unit (ICU) and having blood cultures for the investigation of potential infection was eligible. Patient or consultee agreement was obtained, however patient management was not affected by taking part.

The intention was this study would inform the design of an interventional trial. Between 100 and 120 patients were judged to be sufficient to provide an accurate estimate of effect. Patients were only followed for 5 days after the collection of a blood culture. Given an expected local culture positive rate of 11% at 3 days, around 89% of samples would be expected to be ‘Negative’ on the Cognitor Minus test at day 1.

Patients were monitored during their stay in ICU up to five days from the first blood culture, i.e. to the time of the final blood culture report. On the first day duplicate aerobic (BD BACTEC Plus Aerobic/F Medium (PC: 442192)) and anaerobic (BACTEC Plus Anaerobic/F Medium (PC: 42193)) blood samples were taken for culture. Both sample sets were incubated in the same environment (BD BACTEC FX Blood Culture System). Both sets of blood culture bottles were weighed and the volume of blood added estimated. If there was bacterial growth in any blood culture, the blood was analysed to identify the organism(s) and the results made available for clinical management. The selection of a bottle as test or control within a set was randomised. For test bottles that were negative after at least 12 hours incubation, 0.5 ml aliquots from both aerobic and anaerobic bottles were taken and combined for testing. Test bottles were replaced into BACTEC to complete incubation after aliquots were taken in case growth developed later. The Cognitor Minus test was performed on the combined sample following manufacturer’s instructions but the results were not disclosed. Test results were ‘Negative’ or ‘Not Determined’, the latter meaning presence of organisms was not excluded.

The microbiologist and intensive care consultant or registrar met daily on the critical care units. The intensive care consultant was asked at 24 and 48 hours after the blood culture was taken whether a negative Cognitor Minus result at 99.5% negative predictive value^5^ would have affected their decision to continue, de-escalate or stop antibiotic treatment. The level of confidence and reasons were recorded. The decision if the presence of organism could not be excluded was checked. It was assumed that antibiotic doses could have been saved if the treatment decision following a negative test result went from ‘Continue’/‘Increase’ to ‘Stop’ or from ‘Increase’ to ‘Continue’. When the decision after the test was to ‘Stop’ antibiotics, it was assumed that all doses of antibiotics given from the next day onwards would be saved. As the Cognitor Minus decisions were assessed twice per patient, the clinical decision could differ between days. One decision was chosen based on the relative time of the questionnaires to the time of the Cognitor Minus test (i.e. if the test was run before the first questionnaire, this result was used; however, if run after the first questionnaire, the second was used).

All blood cultures taken in the critical care unit were eligible for inclusion in the study during a period of 6 months. Patients were excluded if under 18 years of age, the treatment intent was palliative, expected discharge from ICU was within 24 hours, or death was imminent. A research nurse recorded demographics, focal signs of infection, temperature, CRP, WBC and antibiotic use plus microbiological cultures for five days. A patient representative helped with designing and writing the study protocol and advised during and after the study.

### Statistical analysis

Continuous variables were summarised by the mean, standard deviation and data range, whilst categorical variables were summarised by the number and percentage in each group. The time from sample incubation to the time of the Cognitor Minus test was described by median and inter-quartile range. The incubation period was theoretically a minimum of 12 hours. The weight of blood in the control and test bottles was estimated from the combined bottle/blood weights. Paired t-test was used to compare blood weight. The five-day blood culture test was used as gold standard for negative predictive values. Confidence intervals were calculated using the exact binomial method. For samples where there was a negative gold standard result at day 5, the characteristics of the true negatives and’Not Determined’ results were compared. Fisher’s exact test was used for the analyses.

The study measured the actual decision on the use of antibiotics, and the decision that would have been reached with a negative result from Cognitor Minus. The percentage of patients in which antibiotics were de-escalated was calculated, along with corresponding confidence intervals (exact binomial method). The level of confidence on the clinical decisions made with and without the Cognitor Minus test result were analysed using the paired t-test.

### Ethics approval and consent to participate

The research was conducted in accordance with the Declaration of Helsinki and national and institutional standards. Approval was obtained from Wales REC 5 198903 16-WA-0264. If the patient had capacity, consent was obtained directly from the patient. However, as is frequent with critical care studies, patients often did not have capacity in which case agreement to participate was obtained either from a personal or nominated consultee. If the patient later regained capacity, retrospective consent was obtained. This was done in line with the Mental Capacity Act 2005. When consent was obtained, it was in the form of written informed consent. The trial was registered with ISRCTN number 17325124 on 7/11/16.

## Results

Over a six-month period, 125 patients were enrolled (Table 1). In 19 other patients, the blood culture became positive within 12 hours or before the test could be run and the patients were excluded from the study. The overall positive blood culture rate was 14.8% (21/141).

**Table 1:** Demographics of the study patients.

Characteristic	Summary
Number of patients	125
Age at admission – Mean ± SD [range]	58 ± 18 [18, 91]
APACHE 2 Score at admission – Median [range]	20 [14–20]
Gender: Female – N (%)	52 (42%)
Male – N (%)	73 (58%)
Admission source: Emergency department – N (%)	33 (26%)
Other hospital – N (%)	13 (10%)
Operating room – N (%)	21 (17%)
Ward – N (%)	58 (46%)
Length of stay days in ICU – (Median Q1, Q3) [range]	11 (7, 21) [2–133]
Severe immunosuppression: Yes – N (%)	92 (74%)
Ventilated: Yes – N (%)	60 (47%)
Vasoactive agents: Yes – N (%)	38 (30%)
Death at ICU discharge: Yes – N (%)	31 (25%)

**Figure 1:**
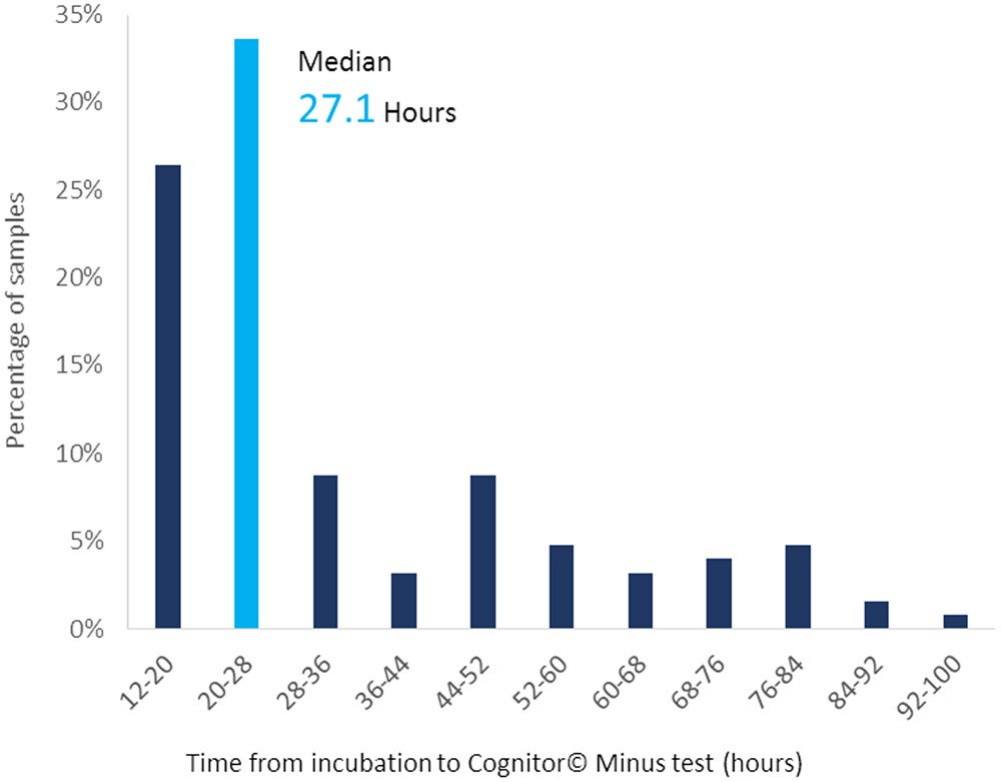
Time from incubation of blood cultures in BACTEC™ to Cognitor© Minus test run in laboratory.

The ward meeting was held between the microbiologist and intensivist at 11:00 each morning and the questionnaires were presented at the first meeting after 12 and 36 hours incubation of the blood culture. The actual results of Cognitor Minus tests were not disclosed. Samples were only collected from the blood culture bottles and processed on the next working day after at least 12 hours incubation. Only bottles showing no growth were sampled. The median time from initial incubation to performance of the Cognitor Minus test was 27.1 hours (IQR 19.6–47.2 h, range 12–91 h) (Fig. 1). Incubation was less than 16 hours for 15 (12%) patients. All tests run after 48 hours incubation reflected blood cultures collected before a weekend or bank holiday. Once removed from the incubator, testing in all cases was started within 20 minutes.

The weight of blood in the bottles was calculated by subtracting the bottle weight (with blood) from the estimated bottle weight from a series of unused bottles (without blood n = 80). Significantly more blood was collected by the clinical staff in the first (control) set (p = 0.03 Table 2). The estimated volumes varied from 0.1 to 23.6 mL. Less than 11% of bottles contained the ideal blood volume of 8–10 mL. Despite randomisation of which bottle was sampled, the amount of the blood in the test bottle was significantly lower than the control bottle for the aerobic samples. On average, there was 0.8 mL less blood in the test bottles. There was no significant difference in blood weights for the anaerobic samples. Fewer control bottles were weighed than test bottles as some were discarded by the routine diagnostic laboratory before they could be weighed.

**Table 2:** Weight of blood in blood culture bottles.

Bottle	N	Mean ± SD	Range	Weight in categories – N (%)		
				<5 mL	5–10 mL	>10 mL
Aerobic control	98	7.8 ± 5.1	0.1–21.6	38 (39%)	29 (30%)	31 (32%)
Anaerobic control	97	7.1 ± 4.3	0.1–21.7	37 (38%)	36 (37%)	24 (25%)
Aerobic test	125	7.1 ± 4.5	0.4–21.5	44 (35%)	55 (44%)	26 (21%)
Anaerobic test	125	6.6 ± 4.3	0.3–23.6	55 (44%)	44 (35%)	26 (21%)

The mean difference in blood weights between Cognitor minus bottles and routine bottles were −0.8 g (95% CI −1.5, −0.1; p = 0.03) for aerobic and −0.4 g (95% CI −1.1, 0.2; p = 0.17) for anaerobic bottles. Some control bottles were discarded by the main hospital laboratory before they could be weighed.

When compared to five-day blood culture results, from the control and test samples, Cognitor Minus demonstrated negative predictive values (NPVs) of 99% and 100% respectively (Table 3). Compared with five-day incubation of control bottles, Cognitor Minus identified 2 of 3 positive blood cultures as result ‘Not Determined’ and one as ‘Negative’ (the bottle sampled for the test was negative, the bottle left in the incubator became positive). Compared with test bottles, all positive blood cultures were identified as ‘Not Determined’. Of 105 ‘Negative’ by Cognitor Minus sets, 48 patients had received broad spectrum antibiotics in the preceding 24 hours compared with all 18 in the ‘Not Determined’ category. With both sets, 18 samples were identified as ‘Not Determined’ of the 122 (routine) and 123 (study) negative blood culture samples.

**Table 3:** Cognitor Minus results from test set compared with results of control and test blood cultures. *Staphylococcus epidermidis in an anaerobic bottle.

Blood culture set	5 day blood culture result	Cognitor Minus result (number of patients)		Negative Predictive Value (NPV, 95% CI)
		Negative	Not Determined	
Control	Negative	104	18	99% (95%, 100%)
	Positive	1*	2	
Test	Negative	105	18	100% (97%, 100%)
	Positive	0	2	

**Figure 2:**
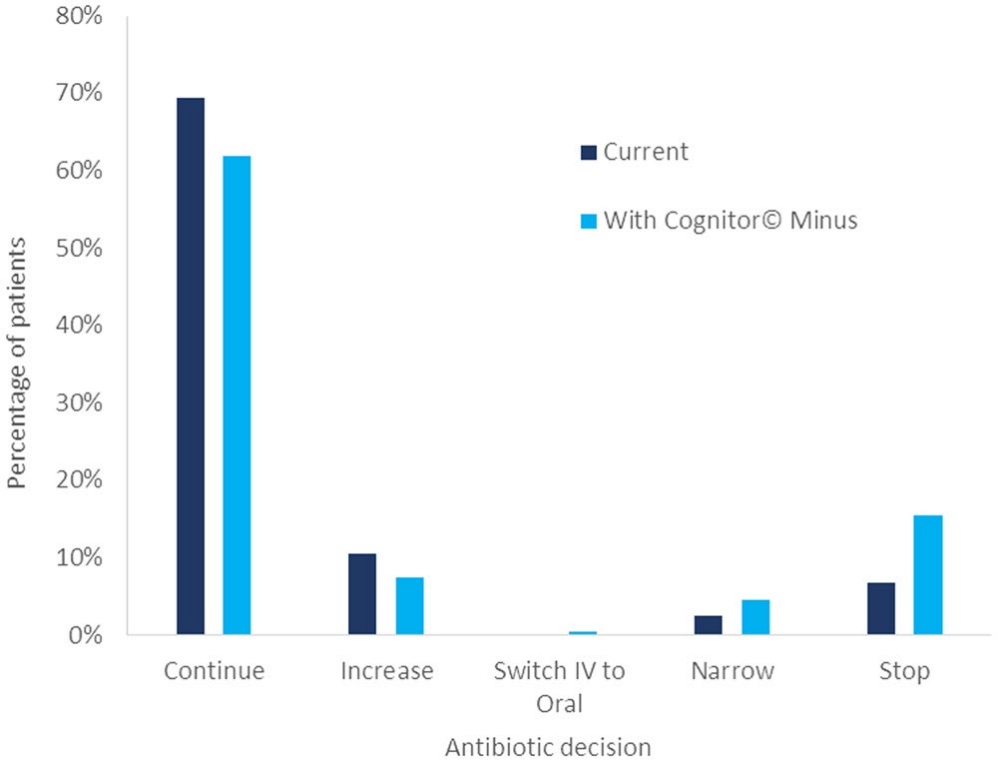
Antibiotic action taken compared with questionnaire decisions on provision of negative Cognitor© Minus test results. Results from first and second questionnaires combined. Patients with no antibiotic treatment not shown.

Actual antibiotic actions were compared with questionnaire results (Fig. 2). Between days 2 and 9 of taking blood cultures, the median length of antibiotic course was 5 days (1–10 days, n = 165). Decisions to ‘Narrow’ or ‘Stop’ were considered de-escalation of antibiotic treatment. Questionnaires suggested that ‘Negative’ Cognitor Minus results would have led to antibiotic treatments being de-escalated in 14% (17/119) (95%CI 9–22%) of patients at 24 and 48 hours when compared with blood culture alone. Antibiotics would have been downgraded for 24% of patients at either or both time points, equivalent to 119 antibiotic doses in 12 patients. There was no significant difference in the level of confidence of the decision with or without the Cognitor Minus result.

No contamination was found in the test bottles when compared with control bottles. With the sample set which had a positive control bottle but negative test bottle, the bottle weight of the control anaerobic bottle indicated a greater volume of blood had been taken compared to the test bottle (6.48 mL vs 1.97 mL) which may have affected the result. The analyses suggested no significant association between the length of the incubation time and a ‘Not Determined’ result.

The weight of blood in both the aerobic and anaerobic bottles was significantly associated with a negative result. For both bottles the general trend was towards a greater amount of blood where there was a ‘Not Determined’ result, and a lesser amount of blood for true negatives (Table 4).

**Table 4:** Comparison of the characteristics of true negative and ‘Not Determined’ (not negative) results.

Variable	Category	True Negative N (%)	Not determined N (%)	P-value
Incubation time	16+ hours	91 (87%)	17 (94%)	0.69
	<16 hours	13 (13%)	1 (6%)	
Blood weight (aerobic)	<5 mL	41 (39%)	2 (11%)	0.02
	5–10 mL	12 (43%)	9 (50%)	
	>10 mL	18 (17%)	7 (39%)	
Blood weight (anaerobic)	<5 mL	51 (49%)	2 (11%)	**0.004**
	5–10 mL	35 (34%)	9 (50%)	
	>5 mL	18 (17%)	7 (39%)	

There were 18 samples where the Cognitor Minus test gave a ‘Not Determined’ result, whilst the blood cultures were negative. Potential pathogens were found to have grown in other clinical samples from 8 of the 18 patients (44%), and were considered at the time of decision making on antibiotic therapy (Table 5).

**Table 5:** Culture results and antibiotic decisions where the Cognitor Minus test result was ‘Not Determined’ (n = 20).

Patient	Microbiology		Antibiotic decision			
	Blood culture (5 days)	Other cultures	Standard Care 1^st^ visit	With Cognitor 1^st^ visit	Standard Care 2nd visit	With Cognitor 2nd visit
18 patients	No growth	Sputum (6/18 patients): *Haemophilus influenzae* *Klebsiella pneumoniae* *Candida albicans* *Candida tropicalis* *Aspergillus sp*. *Staphylococcus aureus* Wound (2/18 patients): *Pseudomonas aeruginosa* *Enterococcus sp*.	Continue: 10/18 Increase: 4/18 Narrow: 1/18 No antibiotics: 2/18 Stop: 1/18	Continue: 12/18 Increase: 1/18 Narrow: 1/18 No antibiotics: 2/18 Stop: 2/18	Continue: 13/18 Increase: 2/18 Narrow: 0/18 No antibiotics: 0/18 Stop: 3/18	Continue: 7/18 Increase: 1/18 Narrow: 1/18 No antibiotics: 0/18 Stop: 8/18
CM014	*Staphylococcus epidermidis* *Candida parapsilosis*	Wound: *Candida parapsilosis*	Continue	Continue	Increase	Continue
CM050	*Candida albicans*	Urine: *Candida albicans* Sputum: Methicillin resistant *Staphylococcus aureus* (MRSA)	Continue	Continue	Increase	Increase

## Discussion

The study suggested that antibiotics would be de-escalated in 24% of patients receiving antibiotic therapy in the critical care unit if the Cognitor Minus result was available at the time of the Microbiology ward round. The test could assist antimicrobial decision-making and lead to a reduction in length of antimicrobial treatment. Reducing doses would have potential cost savings and reduce the risk of antimicrobial resistance emerging. However, in practice, transport delays and scientist working hours could significantly affect the time between sample collection and processing the test^6^. Positive culture results would usually become available at 48 hours but may be negative if taken after antibiotics were started or if the organism is difficult to grow or present in low numbers. Narrowing antibiotic spectrum in patients without a pathogen or antimicrobial susceptibility is usually delayed at least five days, the time at which blood culture is confirmed negative. In a previous observational study using the ETGA (Cognitor Minus) technology on 246 sequential blood cultures negative at 12 hours (n = 197), results were given to the clinical team making treatment decisions^4^. The test result had a positive stewardship outcome in 145 (73.6%) of cases (supporting decision to stop, switch from intravenous to oral or to discharge the patient). However, there was no control group and the rate of positive blood cultures was lower than anticipated.

There was no evidence of low ascertainment of bacteraemia from routine blood cultures samples during this period and no change in processing methods. Under-filling of blood culture bottles was observed, associated with collection of two sets of blood cultures, instead of one, in often difficult circumstances (e.g. hypotension) in critically ill patients and has been observed elsewhere^7^. Only 2.4% of blood cultures in the study showed bacterial growth because blood cultures that became positive within 12 hours of incubation were excluded from the study. However, overall detection rate was as anticipated. Bacteremia surveillance in this unit at the time of the trial showed 10.5–11.3 positive blood culture per 1000 patient-days (63/486 12.3% blood cultures) (national 9.6–10.9) (Infection in Critical Care Quality Improvement Programme, Public Health England personal communication). Nevertheless the focus of this study was negative rather than positive cultures and the effect a negative result had on antibiotic choices.

Cognitor Minus is designed for use on blood samples negative at the time of the test but following at least 12 hours incubation. The test uses Enzymatic Template Generation & Amplification (ETGA) to detect microbial DNA polymerase activity common to a wide range of bacteria and fungi. It produces a high negative predictive value (99.5%)^5^ but a positive value cannot reliably be reported as due to infection. At the time of the test, blood culture bottles must be negative according to automated blood culture following 12 h incubation.

The standard duration of antibiotics was 5 days, although longer courses are used in certain infections, such as osteomyelitis, abdominal sepsis and endocarditis^2^. However, the additional Cognitor Minus result may assist discontinuation of antibiotic in a patient where clinical response is unclear. PCR-based methods of rapid detection identify some blood cultures as containing bacterial DNA without growth as they detect dead organisms or fragments^8^. The group of patients where a confirmation of a negative blood culture may affect the treatment decision would be those where other foci of infection had been eliminated but it was uncertain if active infection remained on the basis of clinical signs. Haematology patients are often the most difficult to determine when to stop antibiotics and a high degree of confidence in the decision is needed. The level of confidence, and the factors recorded as contributory, was the assessment of the clinician at the time of the ward round and was recorded by the research nurse who remained independent.

Sepsis has become a focus of improvement programs with respect to rapid administration of antibiotics within one hour of diagnosis to minimise mortality rates. However, less attention has been placed on the overuse of antibiotic, which results in emergence of bacterial resistance and disruption of normal bacteria flora. De-escalation as part of a stewardship programme does not affect rate of mortality or recurrence rates^1^. In a retrospective study in a critical care unit of patients with bacteraemia, de-escalation of antibiotic treatment reduced the duration of treatment and length of hospital stay^9^. In particular, use of carbapenem was reduced. Rapid identification techniques such as MALDI-TOF can give some guidance to early antimicrobial therapy. When used with antimicrobial stewardship programs, time to appropriate therapy may be improved. In a retrospective study, after implementation of MALDI-TOF identification, time was reduced from 32 to 6.5 hours and susceptibility results from 48 hours to 23 hours^10^. The time to treatment adjustment was reduced from 75 hours to 30 hours (p < 0.001) and costs were significantly reduced. However, a recent prospective double-blind trial suggested that early identification by MALDI-TOF did not affect mortality and might increase the time to appropriate therapy (RAPIDO) (Prof A. MacGowan personal communication).

Tests to exclude bacteraemia in critically ill patients are potentially helpful in reducing the duration of antimicrobial treatment in support of stewardship programmes. However, these are dependent on support given to staff to change treatment outside of the working day.

## Data Availability

The datasets used and/or analysed during the current study are available from the corresponding author on reasonable request.
